# Fabricated devices for performing bacterial-fungal interaction experiments across scales

**DOI:** 10.3389/fmicb.2024.1380199

**Published:** 2024-08-07

**Authors:** Julia M. Kelliher, Leah Y. D. Johnson, Aaron J. Robinson, Reid Longley, Buck T. Hanson, Guillaume Cailleau, Saskia Bindschedler, Pilar Junier, Patrick S. G. Chain

**Affiliations:** ^1^Bioscience Division, Los Alamos National Laboratory, Los Alamos, NM, United States; ^2^Laboratory of Microbiology, Institute of Biology, University of Neuchâtel, Neuchâtel, Switzerland

**Keywords:** bacterial-fungal interactions, BFI, microbial interactions, fabricated devices, microbiology, environmental microbiome

## Abstract

Diverse and complex microbiomes are found in virtually every environment on Earth. Bacteria and fungi often co-dominate environmental microbiomes, and there is growing recognition that bacterial-fungal interactions (BFI) have significant impacts on the functioning of their associated microbiomes, environments, and hosts. Investigating BFI *in vitro* remains a challenge, particularly when attempting to examine interactions at multiple scales of system complexity. Fabricated devices can provide control over both biotic composition and abiotic factors within an experiment to enable the characterization of diverse BFI phenotypes such as modulation of growth rate, production of biomolecules, and alterations to physical movements. Engineered devices ranging from microfluidic chips to simulated rhizosphere systems have been and will continue to be invaluable to BFI research, and it is anticipated that such devices will continue to be developed for diverse applications in the field. This will allow researchers to address specific questions regarding the nature of BFI and how they impact larger microbiome and environmental processes such as biogeochemical cycles, plant productivity, and overall ecosystem resilience. Devices that are currently used for experimental investigations of bacteria, fungi, and BFI are discussed herein along with some of the associated challenges and several recommendations for future device design and applications.

## 1 Introduction

Bacteria and fungi are often the dominant constituents in environmental microbiomes and their relationships, referred to as bacterial-fungal interactions (BFI), are increasingly recognized as significant contributors to microbiome and environmental dynamics (Frey-Klett et al., 2011; Deveau et al., 2018; Bergelson et al., 2019; Wagg et al., 2019). However, there is still much to be uncovered regarding the mechanisms underlying BFI, their phenotypic diversity, and their contributions to local habitats (Frey-Klett et al., 2011; Deveau et al., 2018; Robinson et al., 2021). Community-level approaches such as metabarcoding can be employed to identify the microbial composition of environmental microbiomes, yet confidently assessing bacterial-fungal interactions and their functional impacts from these datasets remains a significant challenge (Mandolini et al., 2021). To address this challenge, researchers must employ smaller-scale *in vitro* techniques, and many groups have designed and used a number of experimental devices which allow them to investigate specific questions regarding the nature of BFI across scales.

Conventional co-culture experiments conducted in Petri dishes allow for the initial identification and characterization of some forms of BFI, however, these techniques often lack environmental complexity or conversely, the granularity for looking at micro-scale or specific interaction phenotypes (e.g., investigating bacterial dispersal along fungal hyphae, or “fungal highways”). Fabricated devices designed to investigate BFI at different biological scales, from the cellular to the community level, have allowed researchers to expand upon traditional co-culture assays to isolate phenotypes of interest or study these interactions under conditions that more closely mimic the natural environments in which they occur (Uehling et al., 2019; Zengler et al., 2019; Kuhn et al., 2022). These devices have various advantages and limitations with respect to sample input requirements, production cost, device size, number of replicates, biological scale(s), ability to assess spatial dynamics, downstream analysis capabilities (e.g., ability to perform omics or re-culture), and control over abiotic and biotic parameters of the system. Devices that create simplified systems with fewer biotic and abiotic factors are often useful for elucidating the molecular mechanisms that underlie interaction phenotypes between specific bacteria and fungi. However, the simplified nature in which BFI are investigated in these devices limits their translatability to interaction dynamics in natural ecosystems. Devices that can recreate more complex environments (increased number of abiotic or biotic factors) can capture additional community dynamics which impact the mechanisms of interaction, and provide insights into the interactions in a more natural environmental context. This review examines the use of fabricated devices to study BFI across multiple scales and levels of system complexity, providing representative examples of device application from the single cell to microbiome level, and provides perspectives on the engineering of future devices to advance BFI research. Particularly, this review will focus on examples of devices for studying BFI in rhizosphere and soil systems, however several of these devices can be used for a variety of research applications. As the field of BFI research continues to expand, the development and implementation of fabricated devices will be foundational to assessing BFI applications to diverse sectors including agriculture, biotechnology, and environmental and human health.

## 2 Current applications of fabricated devices to study BFI across levels of system complexity

### 2.1 Applications of devices to investigate single cell and small-scale bacterial-fungal interactions

**Figure 1:**
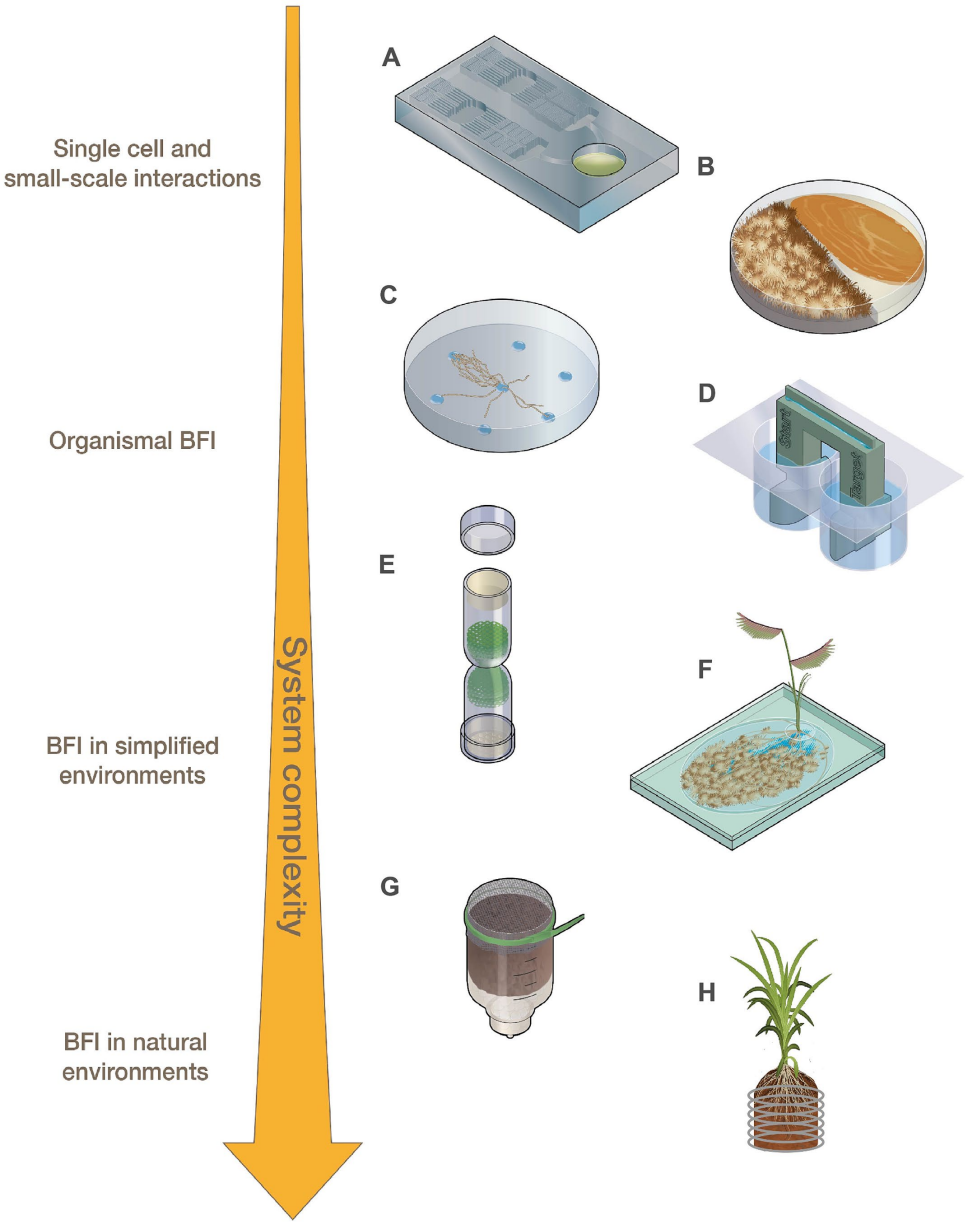
Representations of fabricated devices designed for use across levels of system complexity ranging from small-scale interactions to BFI in natural environments. **(A)** Microfluidic devices, **(B)** modified Petri dishes, **(C)** fungal drops, **(D)** bacterial bridge, **(E)** fungal highway column, **(F)** EcoFAB, **(G)** FlowPot, and **(H)** Rhizogrid.

At the smallest scale, BFI partners are investigated using single-cell or low-biomass approaches. Microfluidic devices (Figure 1A; Table 1), which consist of microchannels on a small chip that are now often constructed using 3D printing, can be applied to small-scale BFI experiments by producing droplet microenvironments for interrogating BFI, or can be designed to investigate BFI dynamics within microscale channels and wells [Grünberger et al., 2014; Stanley et al., 2014; reviewed by Bernier et al. (2022), Masters-Clark et al. (2022), Richter et al. (2022)]. Many microfluidic devices allow for downstream screening and sorting, microscopy-based imaging, and they maintain an extremely controlled environment in terms of both biotic and abiotic variables. Traditional droplet-generating microfluidic devices have been modified to effectively sequester filamentous fungi which can then be screened for specific phenotypes (e.g., enzymatic activity) in a high throughput manner (Beneyton et al., 2016; Samlali et al., 2022). These modified droplet-generating devices can be applied to BFI studies to screen for interaction phenotypes at the cellular level. Niepa et al. (2016) used microfluidic droplets to assess impacts of small signaling molecules on BFI by capturing *Candida albicans* cells alone or together with *Pseudomonas aeruginosa* into these droplets. This study revealed that *P. aeruginosa* was able to impair *C. albicans* growth from both inside (co-captured in droplets) and outside (*P. aeruginosa* in solution surrounding droplets with *C. albicans*) the droplets, indicating that physical interactions are not required to hinder fungal growth and that an exchange of small signaling molecules is likely involved in this antagonistic interaction.

**Table 1:** Applications, features, and considerations for select fabricated devices for BFI research.

Device	Description	Select publications	Features & utility for BFI investigations	Considerations and challenges
Microfluidic devices (Figure 1A)	Chip-based devices that allow for single-cell and small-scale BFI experiments	Stanley et al. (2014), Niepa et al. (2016), Uehling et al. (2019), Gimeno et al. (2021), Arellano-Caicedo et al. (2021), and Bhattacharjee et al. (2022)	Precision control of system with high throughput flexible design that can include many replicates Can be inexpensive and easy to manufacture Can often be directly imaged Invaluable resource for single-cell BFI investigations	Low amounts of recoverable biosample Can be limited by biocompatibility, sterilizability, and transparency of material Can miss interactions that become apparent at larger scales (e.g., organism pigmentation) Can be limited to short term experiments
Modified Petri dish systems (Figure 1B)	Modified Petri dish-based devices enable investigation of specific BFI questions such as structural, spatial, and signaling factors	Álvarez-García et al. (2021), Vlassi et al. (2020), Oosthuizen et al. (2021), and Estoppey et al. (2022)	Allow for whole organism interactions/larger interaction surface area than microfluidics Typically adequate recoverable sample for omics processing Results directly comparable to traditional Petri dish co-cultures	Limited in ability to classify diverse BFI phenotypes and can miss micro-scale details Difficult to scale up to community-level interactions or to add biotic complexity Often lacking heterogeneity seen in soils
Fungal Highway Columns (Figure 1E)	Device for identifying bacterial movement along fungal highways	Kohlmeier et al. (2005), Wick et al. (2007), Simon et al. (2017), and Junier et al. (2021)	Identify and isolate bacteria moving along hyphae Identify fungi creating fungal highways Often include a matrix in center to replicate soil heterogeneity	Limited recoverable biosample (for omics and for re-culturing), especially for bacteria Difficult to image or observe internal matrix
EcoFAB (Figure 1F)	Hydroponic-based device that enables plant-microbiome interaction studies and whole-plant analyses	Gao et al. (2018), Sasse et al. (2019), Zengler et al. (2019), Jabusch et al. (2021), and Novak et al. (2024)	Versatile yet controlled microbial colonization (axenic or designed consortia); can establish controlled root environment (salinity, nutrients) Compatible with imaging techniques and root-system omics Highly reproducible across labs	Not suitable for all plant hosts Limited plant growth window (weeks) Trade-off regarding root growth-chamber matrix (hydroponic maximizes imaging; artificial/natural soil can be used to mimic *in vivo* conditions)
FlowPot (Figure 1G)	Peat-based system that enables host-microbe community investigations	Kremer et al. (2021)	Peat as growth substrate replicates natural soil environments Investigate host-associated interactions and more complex community dynamics with tunable synthetic communities Can apply varied nutrient conditions to the pots	May be difficult to distinguish microbial and host responses (e.g., metabolites) Imaging options may be more limited Optimized for Arabidopsis and may not be suitable for all plants
Rhizogrid (Figure 1H)	3D-printed grids for spatial sampling of plants roots and rhizosphere soils to examine host–microbe interactions	Handakumbura et al. (2021)	Compatible with sterile substrates like sand or natural soil Can customize features such as pots and chamber space to improve plant growth or alter environmental conditions Investigate host-microbe associations with spatial context, e.g., community composition and colonization differences across root sections	May be difficult to distinguish BFI, other microbial community, and host biomolecules/signals, can miss specific interaction dynamics Imaging options may be more limited

To examine how BFI are altered under different nutrient conditions and elucidate the impacts of inter-microbial signals on individual microbes, microfluidic devices have been designed to allow for the addition of media containing specific nutrient profiles, microbial exudates, metabolic products, or small signaling molecules [Groisman et al., 2005; Uehling et al., 2019; reviewed by Richter et al. (2022)]. Uehling et al. (2019) developed a microfluidic device to examine how individual hyphae of *Linnemannia elongata* responded to *Burkholderia*-conditioned media, media conditioned by both the fungal and bacterial partners, and control media. The experiments revealed that the media pre-conditioned by both the fungal and bacterial partners increased fungal growth rates compared to the treatment pre-conditioned with just *Burkholderia*, suggesting that bidirectional BFI communication is key to the observed fungal growth rate modulation. Stanley et al. (2014) designed a microfluidic “fluid exchange” device for media and nutrient exchange during interaction experiments, and it enabled the visualization of physical interactions between *Bacillus subtilis* and *Coprinopsis cinerea* hyphae that were not detectable from Petri dish-based confrontation assays (Schmieder et al., 2019). Microfluidic devices can be constructed with a variety of channel sizes and geometries to assess how microbial interactions and growth are impacted by constraints of their physical environment (Arellano-Caicedo et al., 2021; Gimeno et al., 2021; Mafla-Endara et al., 2021; Bhattacharjee et al., 2022). Arellano-Caicedo et al. (2021) found that microfluidic channel geometry, including channel bend angle, altered hyphal branching patterns which, in turn, impacted bacterial dispersion. Microfabricated ‘soil chip’ devices placed directly into soil enabled researchers to assess how microbes colonize microhabitats, and demonstrated how the presence of fungal hyphae facilitated increased bacterial dispersal through pore spaces (Mafla-Endara et al., 2021). Devices such as this ‘soil chip’ can help to bridge the gap between laboratory *in vitro* experiments and *in situ* environmental experimentation.

### 2.2 Expanding on traditional co-culture assays to understand bacterial-fungal interaction mechanisms at the organismal level

Analysis of bacterial and fungal co-cultures on Petri dishes is one of the most widely utilized methods for characterizing BFI phenotypes. Although not traditionally regarded as fabricated devices, Petri dishes have been modified, for example, by including dividers that split the plates, to help researchers answer BFI-related questions (Figure 1B; Table 1; Vlassi et al., 2020; Álvarez-García et al., 2021; Oosthuizen et al., 2021; Estoppey et al., 2022). Volatile organic compounds (VOCs) are known to play a significant role in many observed BFI, and devices that enable investigations into their roles in microbial interactions are becoming more prevalent [reviewed by Schmidt et al. (2016) and Weisskopf et al. (2021)]. For example, Petri dishes have been altered to physically separate bacterial and fungal cultures to investigate VOCs by allowing the organisms to grow in proximity without physical contact, while maintaining gas exchange (Vlassi et al., 2020; Álvarez-García et al., 2021). Vlassi et al. (2020) identified VOCs generated by the bacteria, *Lysobacter capsici*, that inhibited the growth of known plant pathogenic fungi, *Rhizoctonia solani* and *Sclerotinia minor,* using split Petri dishes (Figure 1B). Other split culture designs allow for the exchange of both volatile and non-volatile compounds while maintaining physical separation of the partners by utilizing membranes with differential permeabilities that exclude microbes from physically interacting but permit the exchange of metabolites such as glycerol and glucose (Oosthuizen et al., 2021).

### 2.3 Devices for investigating hyphal transport of bacteria in simplified environments

Various devices have been developed to interrogate ‘fungal highway’ interactions where bacteria move along or are transported by fungal hyphal networks (Kohlmeier et al., 2005; Warmink and van Elsas, 2009; Simon et al., 2017; Junier et al., 2021). A novel approach to simplifying cell-to-cell observations of BFI was developed in which media droplets with different nutrient profiles are placed on a hydrophobic surface. This ‘fungal drops’ system can be used to assess differences in hyphal sensing and exploration when alone versus when paired with bacterial partners, enabling investigations to determine if bacteria selectively use hyphae as a highway to more nutrient rich areas or to specific nutrients (Figure 1C; Buffi et al., 2023).

Inverted Petri dish-based assays where solid growth media is placed above soil samples have been utilized to identify and isolate soil bacteria capable of traversing fungal hyphae that create bridges between the soil sample and the media (Furuno et al., 2012; Bravo et al., 2013). 3D-printed bacterial bridge and trail devices (Figure 1D) have also been designed to interrogate how bacteria can disperse on a physical scaffold, in a purely abiotic system, to distinguish the contributions of abiotic factors (i.e., liquid film depth and hydraulic flow) from biotic factors (i.e., nutrient exchange and signaling molecules) during hyphal transport of bacteria (Kuhn et al., 2022). Fungal highway columns (Figure 1E; Table 1) are devices that facilitate fungal growth through an inner complex matrix such that bacteria must use hyphae for movement between ends. Various designs of fungal highway columns have enabled insights into how fungal hyphae promote the dispersal and transport of bacteria in simplified communities, soil, dung, and other environmental samples (Kohlmeier et al., 2005; Wick et al., 2007; Simon et al., 2017; Junier et al., 2021).

### 2.4 Devices to mimic the complex natural environments of soil and rhizospheres

To assess the impact of BFI on soil and rhizosphere microbiome dynamics and plant host functioning, experiments can be performed using soil (sterilized or not) in modified microcosms or other devices (Otten et al., 2012; Timm et al., 2016; Zengler et al., 2019; Yee et al., 2021; Aufrecht et al., 2022; Zhang et al., 2023, 2024). The mineral-doped micromodel, a small-scale device designed with a surface mimicking soil texture, porosity, and mineral heterogeneity, was recently developed to understand the role of microbes in mineral weathering. Bhattacharjee et al. (2022) used this device to show that *Fusarium* growth is influenced by the presence of minerals and suggest that the fungus was able to release and uptake the minerals embedded on the chip through indirect weathering. This device can be used to characterize how BFI influence microbial behavior and nutrient acquisition in an environment that simulates simplified soil heterogeneity at small spatial scales.

Devices for investigating impacts of microbial interactions on plants, such as fabricated ecosystems (EcoFABs) (Figure 1F; Table 1; Zengler et al., 2019), Rhizosphere-on-a-chip devices (RhizoChips) (Aufrecht et al., 2022), FlowPots (Figure 1G; Kremer et al., 2021), and Rhizogrids (Figure 1H; Handakumbura et al., 2021), allow for plant growth under hydroponic, synthetic, or natural soil conditions, with some enabling the imaging of root systems and microbial colonization. These devices are also all well-suited for inoculation with synthetic microbial communities. EcoFAB devices (Figure 1F) consist of partitioned root and shoot chambers, which are enclosed to ensure sterile conditions are maintained. The base of an EcoFAB is a large microscope slide, which makes these devices highly compatible with imaging and microscopy systems. EcoFABs have ports which allow for the liquid growth medium to be easily replaced and to facilitate microbial inoculations after plants have established in the device. For example, Coker et al. (2022) generated model synthetic communities representative of diverse grass and crop rhizospheres and examined their compatibility with EcoFAB devices. The researchers showed that these communities were altered in the presence of a plant host (*Brachypodium distachyon*), and demonstrated the ability of community members to colonize roots grown in EcoFABs. RhizoChips are chip-based devices that can be customized with poly-dimethylsiloxane-molded soil structures (Aufrecht et al., 2022). The RhizoChips allow for facile root imaging similar to EcoFABs, but enable spatial sampling to detect relevant plant root exudate hotspots. Adding this environmental and spatial complexity may allow for mechanistic understandings of how natural heterogeneity in nutrient availability impacts BFIs.

FlowPots and Rhizogrids are modified mesocosms that can be inoculated with microbial communities of interest (Handakumbura et al., 2021; Kremer et al., 2021). FlowPots (Figure 1G) are peat-based pots fitted with a mesh cap, which can be flushed with nutrient media for axenic growth, soil slurries to recapitulate a native microbial community, or synthetic microbial communities (Kremer et al., 2021). These pots are grown in gas-permeable boxes to maintain sterile conditions and allow researchers to create a soil-based sterile environment to study how microbial interactions may impact the plant and vice versa under more controlled conditions. Rhizogrids (Figure 1H) are 3D-printed grids which are inserted into a mesocosm or pot (Handakumbura et al., 2021). These grids are disassembled for further experiments such as metabolomics or sequencing and enable researchers to maintain spatial mapping of the source sample based on the section of the grid. BFI partners can be investigated in these systems as individual pairs or as constituents of larger consortia to understand how BFI may impact overall microbial community dynamics or to assess how BFI impact plant hosts (Durán et al., 2018; Marín et al., 2021).

## 3 Discussion

Due to the high diversity and complex nature of BFI, specialized devices are often required to investigate the mechanisms underlying these interactions and their impacts on larger ecosystem processes. The challenges associated with microfluidic device design (e.g., limited environmental complexity, challenges with 3D printing) have been well documented, but many considerations are broadly applicable to other devices (Table 1; Millet et al., 2019; Zhou et al., 2019; Burmeister and Grünberger, 2020). Media type (e.g., liquid vs. solid) and composition (e.g., defined vs. undefined) used within devices can impact microbial growth, alter biomolecule expression, or affect interpretations of resulting omics data (Wang et al., 2012; Basu et al., 2015; Schmidt et al., 2016; Uehling et al., 2019; Black, 2020; Pierce et al., 2021). Devices that can contain multiple media types are therefore highly amenable to BFI investigations as bacteria and fungi can be grown on their preferred media types. While promoting growth for experimental purposes is important for design considerations, how resource availability impacts microbial function and therefore bacterial-fungal interactions has yet to be studied in-depth. Devices that allow for the manipulation of media types or nutrients will additionally enable investigations into how BFIs are impacted by resource availability.

It is known that soil structure impacts microbial community composition and function, which is not captured by most traditional co-culture assays (König et al., 2020; Hartmann and Six, 2023). Materials such as polymer-based substrates, sterilized sand or clay, or glass beads can simulate the complex physical matrix and structure of heterogeneous soil (Nguyen et al., 2005; Droce et al., 2013; Ma et al., 2019; Miebach et al., 2020; Sharma et al., 2020; Del Valle et al., 2022; Rooney et al., 2024). Selecting a device that is compatible with these materials allows for investigations of BFI in microenvironments with more natural physical and spatial characteristics, while providing the means to more easily control biotic and abiotic complexity (Aleklett et al., 2018; Mafla-Endara et al., 2021). For example, synthetic soil-like particles can be added to race tubes, which are traditionally utilized for fungal experiments tracking hyphal growth over time and assessing fungal circadian rhythms. While race tubes have not yet been used to study BFI, this represents an example of how modifications to traditional devices can facilitate enhanced BFI investigations. In general, combining the benefits of fabricated devices with these materials can make BFI investigations more relevant to natural environmental conditions (Simon et al., 2017).

Spatial and temporal dynamics can also be important aspects of experimental design when investigating BFI in fabricated devices, particularly in cases where cells proximal to the interaction may only represent a small fraction of the total biomass. For example, hyphae and cells may have differential molecular responses depending on their proximity to the bacterial partner (Stanley et al., 2014). Therefore, device designs and sampling approaches which permit spatial analyses can be helpful in assessing and normalizing impacts of molecular spatial heterogeneity. Channel sizes in smaller-scale devices may restrict biologically relevant growth, such as hyphal branching, and may quickly become saturated with fungal hyphae, thus limiting temporal studies. Devices that allow for the addition or sampling of partners at different time points may be useful to understand how age, developmental stage, or length of interaction time impact BFI. Many devices can be designed with transparent, biocompatible materials which can be imaged using common microscopy techniques, allowing for the observation of BFI phenotypes over space and time.

When harvesting biomass or biomolecules from devices, it is important to ensure that sufficient material is collected from each partner pair, as BFI experiments are often dominated by the fungal partner in terms of relative biomass. Devices or components that separate fungal and bacterial biomass (e.g., membrane-separated chambers, split Petri dishes, cellophane membranes, etc.) may be desirable for omics-based investigations as these can assist in biomass recovery and allow researchers to more readily determine the origin of signaling or other biomolecules (Guennoc et al., 2017). Microfluidic devices can limit downstream omics-based investigations due to the amount of recoverable or extractable sample material, and may require low-input processing techniques (Tayyrov et al., 2019). Methods to control or measure environmental parameters (e.g., pH, moisture content, temperature) within or around devices are invaluable for monitoring the system and maintaining experimental reproducibility as these variables can alter BFI dynamics or phenotypes (Jiao et al., 2021; Xiong et al., 2021). Implementing the ability to manipulate the environmental conditions of devices will also unlock new research areas to understand how BFIs are impacted by abiotic stress. Understanding how environmental factors influence BFIs is a critical area for understanding how microbial dynamics and their functions shift under both acute and long-term environmental change (e.g., rising temperatures, changes in precipitation). As new materials arise, manufacturing methods progress, and devices become easier and cheaper to fabricate, new biological discoveries will be made at a rapid pace (Niculescu et al., 2021). 3D printing has been popularized due to the availability and relatively low costs of printers and biocompatible materials, as well as the flexibility and customization of device design (Otten et al., 2012; Schunke et al., 2020; Kuhn et al., 2022). Open sourcing of design plans and training in 3D printing software usage help advance devices for BFI investigations by allowing the community to continuously build on past designs. When designing new devices, researchers should consider whether their device can be provided as a resource to the research community, as many devices have been successfully distributed across labs and some have even been used as teaching tools in the classroom (Gao et al., 2018; Millet et al., 2019; Sasse et al., 2019; Junier et al., 2021). Devices with large footprints or high production costs can limit adequate experimental replication or distribution to the community, but may be unavoidable in order to optimize experimental design.

It can be difficult to infer specificity, generality, and causality of functional relationships of interacting microbial partners by only utilizing conventional assays. The continual development of novel devices and techniques for investigating these interactions will lead to more effective functional investigations by optimizing experimental setups and sample harvesting *in vitro*. This review outlines how fabricated devices have been used to study BFI at multiple levels of system complexity, and provides considerations for the use of these devices and the development of future devices. Increased innovation and generation of novel devices will open new research avenues in the rapidly growing field of BFI research and enable exciting new discoveries.

## Author contributions

JK: Conceptualization, Investigation, Supervision, Writing – original draft, Writing – review & editing. LJ: Conceptualization, Investigation, Supervision, Writing – original draft, Writing – review & editing. AR: Conceptualization, Supervision, Writing – original draft, Writing – review & editing. RL: Conceptualization, Writing – original draft, Writing – review & editing. BH: Conceptualization, Supervision, Writing – original draft, Writing – review & editing. GC: Visualization, Writing – original draft, Writing – review & editing. SB: Supervision, Writing – original draft, Writing – review & editing. PJ: Conceptualization, Supervision, Writing – original draft, Writing – review & editing. PC: Conceptualization, Funding acquisition, Project administration, Supervision, Writing – original draft, Writing – review & editing.
